# Systemic pirfenidone treatment fails to mitigate fibrosis and compromises functional recovery in a porcine model of volumetric muscle loss

**DOI:** 10.1113/EP093693

**Published:** 2026-07-30

**Authors:** Jessica M. Motherwell, Ondine Eken, Claudia E. Hernandez, Christopher L. Dearth, Stephen M. Goldman

**Affiliations:** ^1^ Extremity Trauma and Amputation Center of Excellence Defense Health Agency Falls Church Virginia USA; ^2^ Department of Surgery Uniformed Services University of the Health Sciences Bethesda Maryland USA; ^3^ The Henry M. Jackson Foundation for the Advancement of Military Medicine Inc. Bethesda Maryland USA; ^4^ C2 Alaska LLC San Antonio Texas USA

**Keywords:** fibrosis, inflammation, regeneration, trauma, wound healing

## Abstract

Repurposing clinically approved antifibrotic agents presents a potential near‐term therapeutic strategy to mitigate the extensive fibrotic scarring that follows severe volumetric muscle loss (VML) injury and inhibits functional recovery. We hypothesized that daily oral administration of pirfenidone, an FDA‐approved antifibrotic, would mitigate the overwhelming fibrotic response and facilitate functional recovery in a clinically relevant porcine VML model. Yorkshire‐cross pigs (*n* = 12) underwent VML or sham surgery in the peroneus tertius muscle and were subsequently allocated to pirfenidone treatment or untreated controls. At the 4‐week study endpoint, the affected muscles underwent comprehensive histological, biochemical, transcriptional and neuromuscular functional assessments. Transcription profiling demonstrated that pirfenidone downregulated key pro‐inflammatory cytokine and chemokine regulators involved in excessive fibrosis, including TNF, CCL3L1 and CCL2. While pirfenidone treatment failed to reduce total collagen deposition, evidenced by picrosirius red quantification and hydroxyproline content, it did result in a less dense collagen fibre architecture, suggestive of altered tissue remodelling. However, pirfenidone treatment impaired functional recovery through reduced contractile output and resulted in stagnant weight gain compared to the untreated cohort. This work demonstrates that systemic pirfenidone administration failed to meaningfully reduce fibrosis and subsequently worsened functional capacity following VML, similar to previous antifibrotic studies. The functional deficits and stagnant weight gains observed here underscore a critical knowledge gap regarding the impact of systemic antifibrotics on contractile recovery, necessitating a rigorous re‐evaluation of delivery routes, dosages and administration timelines to ensure that future therapies for fibrotic scarring do not inadvertently hinder the physiological recovery of VML‐injured muscle.

## Abstract (highlights)

- **What is the central question of this study?**
  Does administration of pirfenidone elicit an antifibrotic response following volumetric muscle loss (VML) injury?
- **What is the main finding and its importance?**
  Systemic pirfenidone administration failed to reduce total collagen in a clinically relevant VML model, despite modulating collagen fibre architecture, and impaired muscle function. Pirfenidone treatment was insufficient to overcome the fibrotic response and proved detrimental to muscle recovery. These findings, consistent with prior research, highlight the need to re‐evaluate antifibrotic delivery strategies to ensure that scar‐mitigation efforts do not inadvertently compromise the contractile restoration of VML‐injured muscle.

## 1 INTRODUCTION

Volumetric muscle loss (VML) is a debilitating condition arising from traumatic injury or surgical procedure (e.g. cancer resection) that results in a loss of functional skeletal muscle that exceeds the endogenous capacity of the affected musculature to functionally regenerate (Grogan et al., 2011). This pathology is characterized by a persistent inflammatory response, which promotes excessive fibrotic deposition within the VML defect that is primarily driven by the transforming growth factor‐β1 (TGF‐β1) pathway (Aguilar et al., 2018; Corona et al., 2016; Garg et al., 2015). The interplay between pro‐inflammatory and pro‐fibrotic signalling establishes a feedback loop that contributes to the inhibition of *de novo* myogenesis, thereby preventing functional recovery of the injured muscle (Aguilar et al., 2018). These pathological processes lead to impaired physiological function, manifesting clinically as reduced range of motion and loss of neuromuscular control.

Previous VML research studies have highlighted the need for therapeutic interventions aimed at reducing fibrotic scarring to improve the microenvironment for tissue regeneration. A potential near‐term strategy is to repurpose clinical treatments for similar fibrotic pathological conditions, such as idiopathic pulmonary fibrosis (IPF). Currently there are three FDA‐approved medications for the treatment of IPF: nintedanib, pirfenidone and the recently approved nerandomilast (Hujjat et al., 2026; Ratner, 2014). Nintedanib, a tyrosine kinase inhibitor that targets growth factor receptors, attenuated fibrosis in a porcine model of VML, however the intervention also reduced the functional capacity of the injured musculature (Corona et al., 2020). Given this outcome and the recent market entry of nerandomilast, pirfenidone represents a more established candidate for reducing fibrotic deposition. Pirfenidone is an orally administered small molecule with antifibrotic properties that act through suppression of the TGF‐β1 pathway and modulation of pro‐inflammatory cytokine production (Conte et al., 2014; Ma et al., 2018; Ruwanpura et al., 2020). With successful outcomes in slowing the progression of IPF and widespread clinical use, researchers have investigated alternative applications for pirfenidone to target similar pathological scarring conditions across hepatic, renal and cardiac tissues (Qin et al., 2018; Schaefer et al., 2011; Van Erp et al., 2006). Given its proven clinical utility and effectiveness in several animal models, pirfenidone treatment is a promising candidate for modulating the TGF‐β1 pathway to reduce fibrosis in musculoskeletal trauma. Repurposing established clinical treatments to target the complex pathology of VML represents a viable strategy to facilitate functional restoration and improve the quality of life for patients. Thus, leveraging the FDA‐approval status for near‐term translation, we investigated whether administration of pirfenidone would elicit an antifibrotic response following VML injury.

In this study, we used a clinically relevant porcine animal model of VML to test the hypothesis that daily pirfenidone administration, initiated immediately following injury, would mitigate pro‐fibrotic mediators, thereby promoting functional skeletal muscle recovery. To evaluate these effects, we performed biochemical, histological and in vivo assessments of muscle function at 4 weeks post‐injury.

## 2 METHODS

All experiments were conducted in compliance with the Animal Welfare Act, the Implementing Animal Welfare Regulations, the principles of the *Guide for the Care and Use of Laboratory Animals*, and the Animal Research: Reporting of In Vivo Experiments (ARRIVE) guidelines. Procedures and care guidelines were approved by the Institutional Animal Care and Use Committee at the Uniformed Services University of the Health Sciences (Protocol no. SUR‐23‐127; USUHS; Bethesda, MD, USA). The investigators understand the ethical principles under which *Experimental Physiology* operates and confirm that all experimental protocols are in compliance.

### 2.1 Animal preparation and monitoring

All animals were housed in standard runs and exposed to a 12‐h light/dark cycle, with ad libitum access to water and twice‐daily feedings. Twelve Yorkshire‐cross male pigs (34.3 ± 2.4 kg; ∼12 weeks old; Midwest Research Swine; Glencoe, MN, USA) were block randomized to either an untreated control group or a pirfenidone‐treated cohort (*n* = 6 animals per group) (Figure 2a). Animals were fasted overnight prior to the day of VML procedure and the study endpoint of 4 weeks, with ad libitum access to water. The animals were pre‐medicated with an intramuscular injection of Telazol–xylazine (4.4 mg/kg and 2.2 mg/kg), and induction of anaesthesia was performed with 1% isoflurane by face mask. Orotracheal intubation was performed using an endotracheal tube and animals were placed on an automatic ventilator to maintain end tidal $P_{CO_{2}}$ at 40 ± 5 mmHg, with general anaesthesia achieved by isoflurane (1–3%). Animals were monitored throughout the procedure to assess for anaesthetic depth and vital signs. Once an adequate depth of anaesthesia was determined, vascular access was established with a catheter secured in the ear vein to administer hydrating fluids. Prior to the VML procedure, animals received buprenorphine SR (0.24 mg/kg) by subcutaneous injection. At the study endpoint, immediately following the final neuromuscular functional assessments, animals were humanely euthanized with an intravenous injection of Euthasol (1 mL/4.5 kg body weight; Virbac AH Inc.; Westlake, TX, USA) while under anaesthesia. Peroneus tertius (PT) muscles of the hindlimbs were then harvested and sliced into multiple cross‐sections taken at the VML injury site for biochemical and histological analyses.

### 2.2 VML procedure

All animals underwent a unilateral VML procedure in the PT muscle, while the contralateral hindlimb received a sham operation to serve as an internal, uninjured control. This experimental design resulted in 12 total limbs per group for subsequent functional analysis, partitioned into six VML‐injured and six sham‐operated. The procedure for creating a unilateral defect was performed based on a well‐characterized porcine model of VML injury in the anterior compartment of the lower hindlimbs (Corona et al., 2018, 2017; Greising et al., 2017; Ward et al., 2016). Using sterile technique, a full thickness VML defect was created by excising a target of 5 g of tissue from the belly of the PT muscle. The contralateral limb underwent a sham surgical procedure as previously described (Corona et al., 2020). Wounds were closed by suturing the fascia and skin in individual layers, and a topical antibiotic ointment (bacitracin zinc/neomycin sulfate/polymyxin B sulfate) was applied to the closure. Following surgery, all animals were monitored twice daily for three days to assess any signs of complications with wound closure, dehydration and pain management.

### 2.3 Pirfenidone treatment

Immediately following VML injury, animals receiving pirfenidone (Esbriet, Genentech, Inc.; San Francisco, CA, USA) began treatment according to the clinical dosing schedule for 267 mg tablets (LegacyPharma, 2017). For the first week, animals received one tablet three times daily, for a total of 801 mg per day. The second week, animals received two tablets three times daily, for a total of 1602 mg per day. For the third and fourth weeks, animals received three tablets three times daily, for a total of 2403 mg per day, which is the human equivalent dose recommended for treating IPF. The dosing schedule is required to allow the body to gradually adjust to the medication and minimize gastrointestinal side effects (LegacyPharma, 2017).

### 2.4 In vivo neuromuscular assessments and passive tension

In vivo neuromuscular functional capacity was assessed in the lower hindlimbs pre‐ and post‐operatively to assess baseline strength of the dorsiflexor muscles in their uninjured and acutely injured state, respectively. These assessments were repeated again at the study endpoint of 4 weeks post‐injury. Functional measurements were performed on the dorsiflexor muscles of the anterior compartment in VML injured and sham‐operated hindlimbs of anaesthetized pigs using a large animal force transducer (Model 890A, Aurora Scientific Inc.; Aurora, Canada). The knee was positioned at a 90° angle, and the hoof was positioned in a 25° plantar‐flexed position (Figure 2e). Transcutaneous electrodes were inserted on either side of the peroneal nerve, and the optimal stimulation voltage was determined for each animal with a series of twitch and tetanic contractions (150 Hz, 0.1 ms pulse width, 800 ms train). Passive tension was assessed over a range of joint angles (90°–140°) by measuring the resting torque (i.e., resisting stretch) and normalized to endpoint body weight. Subsequently, tetanic torque measurements were taken across a range of frequencies (10–200 Hz) for force–frequency analysis. At the 4‐week study endpoint, passive tension and force–frequency measurements were first assessed, followed by a tenotomy on the distal tendons of the synergist dorsiflexor muscles to acquire maximum tetanic force on the isolated PT muscle.

### 2.5 Transcriptional analysis

A sample of the PT muscle taken within the defect region was collected at the study endpoint and snap frozen in liquid nitrogen. Samples were subsequently homogenized, and RNA was isolated from tissue samples using QIAzol and miRNeasy Mini kits. The RNA samples were reverse transcribed into cDNA using the RT2 First Strand kit. Gene expression was analysed using gene arrays (PASS‐120Z, RT2 Profiler PCR Array; Qiagen Sciences, Inc.; Germantown, MD, USA). All protocols followed the manufacturer's specifications. Fold change for gene targets was normalized to sham‐operated limbs in the untreated group prior to analysis.

### 2.6 Hydroxyproline assay

A sample from the defect region of the PT muscle was collected at the study endpoint, frozen in liquid nitrogen and stored at −80°C until analysis. Tissue samples were lyophilized and measured for final dry muscle weight. Dried muscles were processed using a papain buffer (Sigma‐Aldrich, Saint Louis, MO, USA) until fully digested, then hydrolysed in 12 M HCl at 100°C for 3 h and 95°C for 16 h. Hydroxyproline was measured from the hydrolysate and used to quantify collagen content. Briefly, hydrolysed samples and hydroxyproline standard (100 µg/mL) were incubated with chloramine‐T solution at room temperature for 20 min followed by para‐dimethylaminobenzaldehyde in a water bath heated to 60°C for 30 min. Samples were subsequently measured on a plate reader at 550 nm wavelength. The total collagen content for each muscle sample was quantified by assuming a ratio of 1:12.5 for hydroxyproline to collagen and normalized to dry muscle weight.

### 2.7 Histology

A slice of the PT muscle taken at the defect site was formalin fixed (Sigma‐Aldrich; Saint Louis, MO, USA) and paraffin embedded for histological staining. Tissues were sectioned at a thickness of 7 µm. Sections stained with picrosirius red (PSR) or haematoxylin and eosin (H&E) following the manufacturer's suggested protocol and then sealed with Surgipath Micromounting Medium (Leica; Wetzlar, Germany). PSR‐ and H&E‐stained tissues were imaged at ×10 magnification with an Axio Scan Z1 (Zeiss; Oberkochen, Germany) slide scanning microscope, and acquisition parameters were standardized for all samples. Circular polarized light images of PSR‐stained tissues were acquired at ×10 magnification with an Axiolab 5 microscope (Zeiss; Oberkochen, Germany).

### 2.8 PSR imaging and quantification

Collagen deposition was analysed from PSR‐stained muscle cross‐sections taken at the defect region and quantified as a percentage of total tissue area. Images were processed using ImageJ software, where collagen and tissue area parameters were isolated by colour using the Color Deconvolution and the band‐pass filter Threshold Colour plug‐ins (Landini et al., 2021; Ruifrok & Johnston, 2001; Schindelin et al., 2012). Images were then binarized using a global threshold, and measurements were calculated. Tissue area measurements were calculated from the cross‐section of muscle samples in the binarized image, including muscle fibres and connective tissue. The percentage of collagen per total tissue area was then calculated for each PT muscle. To quantify collagen fibre maturity, PT muscles from VML‐injured limbs (*n* = 6 per group) were imaged at ×20 magnification using circular polarized light microscopy to capture five regions of interest (ROIs) within the injury site. The area of collagen fibres as a function of their colour hue was then quantified, where the hue corresponds to relative fibre thickness from thin green fibres to increasingly thick yellow, orange and red fibres (Cuttle et al., 2005; Dolan et al., 2022; Hoffman et al., 2022; Nadkarni et al., 2007; Wolf et al., 2014). A custom MATLAB (The Mathworks; Natick, MA, USA) script transformed each image from the RGB to the HSV colour model, separated each colour component as a function of hue (red 2–9 and 230–256, orange 10–38, yellow 39–51, green 52–128), applied a threshold to remove noise from an average of a global threshold using Otsu's method (intensity value of 50/256), and expressed the collagen content for densely packed collagen (red, orange, yellow) and loosely packed collagen (green) as a percentage of the area of each image. For each PT muscle (*n* = 6 per group), the five ROIs were averaged together per muscle sample prior to statistical analysis.

### 2.9 Statistical analysis

All data are reported as means ± standard deviation (SD) with statistically significant differences defined at *P* < 0.05. Prior to analysis, all datasets were evaluated for normal distribution and equal variance. Statistical analysis was conducted using GraphPad Prism software version 9.3.1 (GraphPad Software; Boston, MA, USA) or R program (R Core Team, 2025). Data were analysed using two‐way, nested two‐way or three‐way analysis of variance (ANOVA) with Šidák's *post hoc* test, used as appropriate; Student's unpaired two‐tailed *t*‐test was employed for single‐point comparisons when evaluating VML‐injured limbs between pirfenidone treated and untreated groups.

## 3 RESULTS

PCR arrays were used to determine whether pirfenidone treatment reduced pro‐fibrotic genes at the transcription level at 4 weeks following VML injury. The gene targets were normalized to the sham‐operated muscles from the untreated cohort (sham‐untreated) prior to comparison between VML‐untreated and VML‐pirfenidone limbs. Of the 83 genes evaluated, only three were found to be downregulated in the pirfenidone group compared to untreated (Figure 1a). These genes were tumour necrosis factor (*TNF*), C‐C motif chemokine ligand 3 like 1 (*CCL3L1*), and monocyte chemotactic protein‐1 (*CCL2*) (Figure 1b). Additional genes that were downregulated, but less than 2‐fold change were tissue inhibitor of metalloproteinase 3 (*TIMP3*), interleukin‐13 receptor subunit alpha 2 (*IL13RA2*), and eotaxin‐1 (*CCL11*) (Figure 1c). No genes were found to be upregulated by pirfenidone treatment. Qualitative assessments of the VML injury revealed no differences in fibrosis at the defect site between pirfenidone and untreated muscles (Figure 1d). This finding was further supported by quantification of collagen deposition, measured as a fraction of anatomical cross‐sectional area, where we found no reduction in fibrosis attributed to pirfenidone treatment in VML‐injured muscles (*P* = 0.0849) (Figure 1e). Analysis of PSR‐stained VML injuries under polarized light indicated that collagen fibre distribution varied significantly based on both treatment and fibre density (interaction; *P *< 0.0001) (Figure 1g). Further comparisons showed that pirfenidone treatment resulted in a defect site with increased loosely packed (green) fibres (91.4%; *P* = 0.0007). Conversely, untreated muscles possessed a greater frequency of densely packed (red/orange/yellow) collagen fibres (*P* = 0.0604). Similarly, a hydroxyproline assay, used as a measure to determine collagen content in VML‐injured muscles, found no differences between pirfenidone or untreated groups (*P* = 0.5291) (Figure 1f). Further quantification of the muscle anatomical cross‐sectional area found no differences resulting from pirfenidone treatment (*P* = 0.0936) (Figure 1h). We did not observe any differences in morphological remodelling from qualitative assessments of H&E‐stained muscle cross‐sections. Muscles from both groups showed infiltrating cells around the border of the defect, along with remodelling muscle fibres characterized by centrally located nuclei (Figure 1d).

**FIGURE 1:**
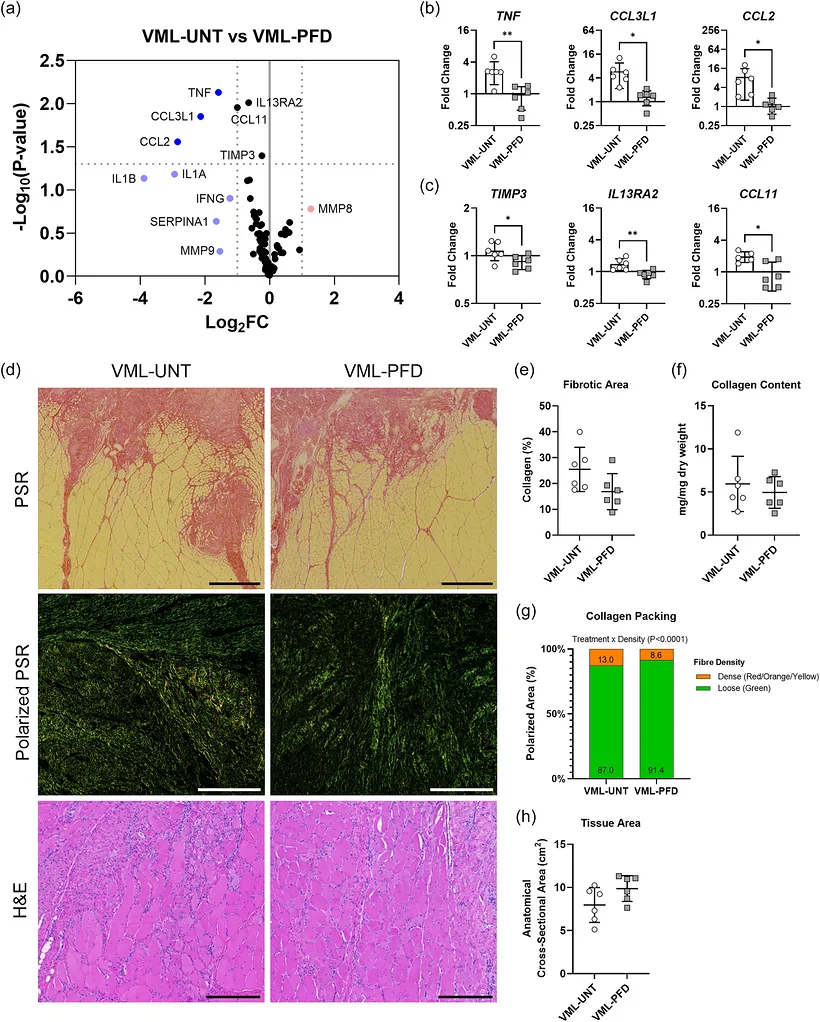
Molecular and histological assessments of fibrotic deposition and morphological remodelling following pirfenidone treatment in VML‐injured muscles. (a) Volcano plot illustrating differentially expressed genes in pirfenidone‐treated (VML‐PFD) versus untreated (VML‐UNT) muscles at 4 weeks post‐injury. Blue and red points denote significantly downregulated and upregulated genes, respectively. (b, c) Fold change of select downregulated genes categorized by significant downregulation (*P *< 0.05, log2 fold change (FC) > 1) (b) and significance but less than 2‐fold (*P *< 0.05, log2FC < 2) (c) relative to the VML‐UNT group. (d) Representative histological sections showing PSR staining for total collagen (top; scale bar = 1000 µm), polarized light microscopy of PSR‐stained sections to evaluate fibre maturity (middle; scale bar = 300 µm), and H&E staining for morphological assessment (bottom; scale bar = 300 µm). (e) Quantification of percent fibrotic area from PSR‐stained cross‐sections. (f) Muscle collagen content normalized to dry weight, as estimated by hydroxyproline analysis. (g) Distribution of collagen fibre maturity (thin to thick) based on polarized PSR light analysis. (h) Quantification of anatomical cross‐sectional tissue area (cm^2^). Data are presented as means ± SD; *n* = 6 muscles per group. Statistical significance for (b, c, e, f, h) was determined by two‐tailed unpaired *t*‐test. For (g), statistical differences were evaluated by a nested two‐way ANOVA followed by Šidák's *post hoc* comparison. Significance is indicated by asterisks: *P < 0.05, **P < 0.01. VML‐UNT: VML‐injured untreated muscles; VML‐PFD: VML‐injured pirfenidone‐treated muscles.

Pirfenidone‐treated animals exhibited less weight gain throughout the study, resulting in a terminal body weight that was 24% lower than untreated controls (Figure 2b). VML‐injured PT muscle wet weights were lower compared to sham‐operated (main effect; *P* = 0.0009); however, no differences were observed by pirfenidone treatment (Figure 2c). To account for variations in weight gain, PT muscle mass was normalized to terminal body weight. Analysis revealed significant main effects for both pirfenidone treatment (*P* = 0.0003) and VML injury (*P* = 0.0017), both of which significantly reduced the muscle‐to‐body‐weight ratio relative to the untreated group. Specifically, both VML‐injured (*P* = 0.0002) and sham‐operated (*P* = 0.0004) limbs exhibited lower relative normalized mass than untreated controls (Figure 2d). Muscle stiffness, as determined by passive torque, increased significantly with joint angle in VML‐injured limbs compared to sham controls (main effect; *P* = 0.0067). No significant impact was attributed to pirfenidone treatment (Figure 2f). To assess functional recovery, dorsiflexion torque was recorded prior to injury, immediately post‐injury and at 4 weeks. VML injury resulted in a significant temporal decline in contractile output (interaction; *P* = 0.0004). Further analysis confirmed that while no differences existed prior to injury (*P *> 0.9999), muscle strength was significantly decreased both immediately following the procedure (*P* = 0.0027) and at the study endpoint (*P* = 0.0002) in VML‐injured limbs (Figure 2h). Assessment of the torque–frequency relationship revealed a VML‐induced deficit in contractile capacity at the study endpoint. Analysis of derived parameters – including twitch, half‐max, Hill slope, and tetanic force – confirmed that while VML injury impaired overall function, pirfenidone treatment further exacerbated this decline as evidenced by normalization to endpoint PT muscle mass and absolute torque values (Figure 2*i,l,o*). To isolate the PT muscle, a tenotomy was performed and maximum torque measurements were subsequently collected. When normalized to terminal body weight, VML injury significantly reduced torque (main effect; *P* = 0.0040), though no differences were observed between the pirfenidone‐treated and untreated cohorts. Interestingly, when accounting for muscle size through normalization to PT mass, pirfenidone treatment significantly reduced contractile output (main effect; *P* = 0.0002) overall, with both VML‐injured (*P* = 0.0060) and sham‐operated limbs (*P* = 0.0101) lower than the untreated limbs. Evaluation of absolute torque values similarly identified main effects for both VML injury (*P* = 0.0043) and pirfenidone (*P* = 0.0113), where the antifibrotic treatment significantly compromised force production, particularly within the sham‐operated group (*P* = 0.0467) (Figure 2*k,n,q*).

**FIGURE 2:**
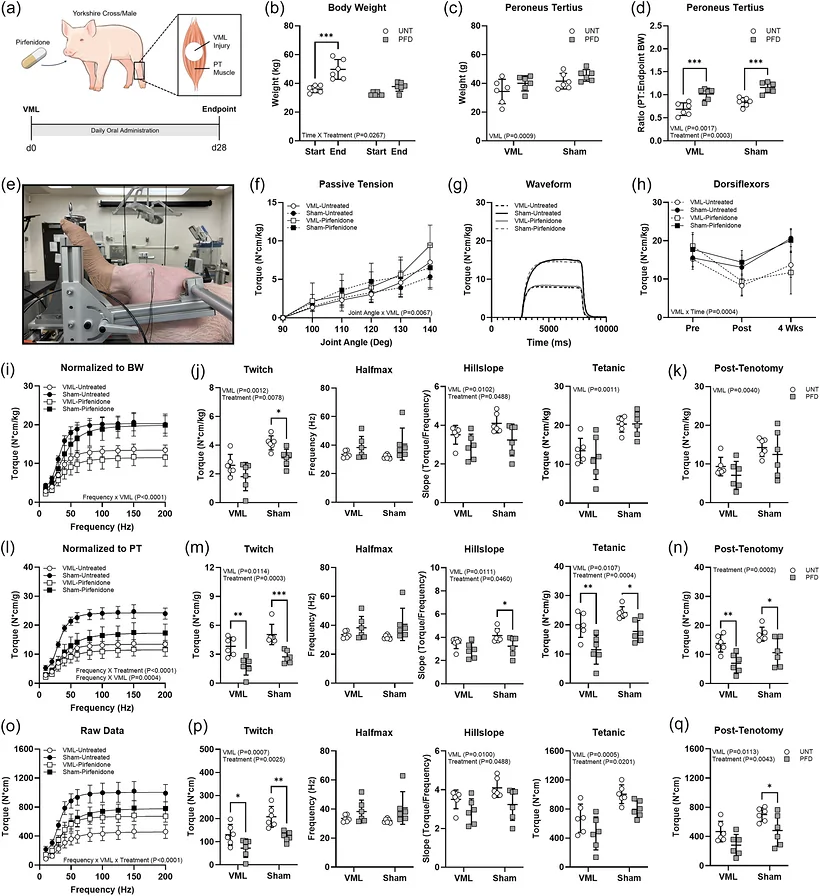
Functional characterization of PT muscle recovery following pirfenidone treatment for volumetric muscle loss. (a) Experimental timeline and schematic illustration of the porcine VML model and daily pirfenidone administration. (b) Longitudinal animal body weight recorded from study initiation to the 28‐day endpoint. (c, d) PT muscle wet weight at 4 weeks post‐injury (c) and the resulting muscle‐to‐terminal body weight ratio (d). (e) Photographic representation of the in vivo neuromuscular functional assessment set‐up. (f) Plantar flexion about the ankle joint collected as a measure of muscle stiffness at 4 weeks. (g) Representative trace of peak isometric torque measurements from VML‐injured and sham‐operated limbs. (h) Longitudinal evaluation of dorsiflexion torque at baseline (pre‐injury), immediately post‐injury, and at the 4‐week endpoint. (i–q) Comprehensive contractile analysis presented as force–frequency curves (i, l, o), derived parameters (twitch, half‐maximal frequency, Hill slope and tetanic torque) (j, m, p), and isolated PT torque recorded post‐tenotomy (k, n, q). Data are categorized by normalization method: relative to terminal body weight (i–k), relative to PT muscle mass (l–n), and absolute raw torque (o–q). Data are presented as means ± SD; *n* = 6 muscles per group. For (b, c, d, j, k, m, n, p, q), statistical significance was determined by two‐way ANOVA with Šidák's *post hoc* test. For (f, h, i, l, o), a three‐way ANOVA was utilized. Significance is indicated by asterisks: *P < 0.05, **P < 0.01, and ***P < 0.001. Illustrations were provided by Servier Medical Art, licensed under CC BY 4.0 and NIAID NIH BioArt Source.

## 4 DISCUSSION

This study provides evidence that pirfenidone downregulated fibrotic mediators through inhibition of key inflammatory signalling and the modulation collagen fibre density. Conversely, the treatment also resulted in suboptimal functional outcomes. These data suggest that pirfenidone treatment, as administered here, negatively impacts the physiological recovery of VML‐injured muscle. The administered dose was chosen based on established clinical guidelines for treatment of IPF in adults and has shown efficacy in a large animal model of congestive heart failure (Kim & Keating, 2015; Lee et al., 2006). However, the current dosing regimen does not provide a clinically meaningful therapeutic benefit with pirfenidone as a strategy for mitigating fibrotic scar formation in VML.

Herein, we administered pirfenidone for one month immediately following VML injury, a time period sufficient for chronic inflammation and fibrotic scarring to develop. Persistent inflammation is a defining feature of the fibrotic process in VML, where the extended presence of inflammatory cells drives the continuous deposition of extracellular matrix (ECM), leading to detrimental fibrotic scarring and functional decline of the affected musculature (Larouche et al., 2022). Given that chronic inflammation is a hallmark of VML, which contributes to fibrotic tissue development and diminished functional capacity, pirfenidone treatment was hypothesized to help alleviate this cascade (Aguilar et al., 2018; Altamirano et al., 2025; Greising et al., 2017). The lack of reduction in overall collagen content suggests that either the systemic dose administered was insufficient to counteract the aggressive fibrotic response, or that the specific mechanisms targeted by the treatment were unable to overcome the primary drivers of scar formation. Despite pirfenidone having known antifibrotic properties, we did not observe meaningful reductions of fibrotic tissue formation in the present study, supported by transcriptional profiling and direct collagen quantification from both histological and biochemical assays. However, we did observe less densely packed collagen fibres in pirfenidone treated muscles, suggesting an improvement in tissue remodelling at the defect site. Pirfenidone acts by attenuating several pathways involved in fibrosis, most notably TGF‐β1, and this pleiotropic mechanism of action may have been insufficient to achieve a clinically meaningful impact in this model of VML (Ruwanpura et al., 2020). Nintedanib was previously shown to reduce fibrotic markers of VML and muscle stiffness, and interestingly, the study also reported a negative impact on muscle function and terminal body weight following treatment (Corona et al., 2020). We observed a similar reduction in contractile output and lack of weight gain with pirfenidone treatment, although the effect on fibrotic scarring was minimal. Furthermore, we observed functional deficits in the contralateral sham‐operated limbs, suggesting that systemic pirfenidone treatment negatively impacts neuromuscular function outcomes in unaffected muscle as well (Figure 2). These findings indicate a broader complication with targeting fibrotic scarring using systemic antifibrotic treatments, which appear to compromise the functional integrity of VML‐injured limbs, and unaffected musculature in the case of pirfenidone. Nintedanib acts specifically by blocking multiple growth factor receptors, suggesting that a more potent and targeted antifibrotic treatment may be warranted to successfully reduce collagen formation in VML injuries. Nevertheless, the observed functional impairment and stagnant weight gain necessitates a re‐evaluation of antifibrotic treatment for VML repair, specifically regarding the delivery route, therapeutic dosage and duration of administration.

Despite the failure to reduce fibrosis, transcriptional profiling provided evidence that pirfenidone was pharmacologically active within the VML defect and effectively modulated inflammatory mediators associated with fibrosis. Specifically, we observed a reduction in pro‐inflammatory cytokine and chemokine gene transcripts, including *TNF*, *CCL3L1* and *CCL2*. This finding is not surprising given that pirfenidone has anti‐inflammatory properties due to its pleiotropic mechanisms and ability to diminish cytokine production (Hirano et al., 2006; Spond et al., 2003; Toda et al., 2018; Visner et al., 2009). Suppression of these mediators is important, as they contribute to fibrotic scarring through upregulation of ECM components and increased inflammatory cell activity, which collectively creates a tissue microenvironment that obstructs muscle repair (Aguilar et al., 2018; Larouche et al., 2022). While pirfenidone did not achieve its intended primary outcome in this study, these anti‐inflammatory properties demonstrate utility in combating the chronic inflammation that accompanies VML injuries, thereby supporting further exploration as an adjunct treatment in a combination therapy.

In summary, this study demonstrated that systemic administration of pirfenidone proved insufficient to reduce fibrotic scar formation in a clinically relevant model of VML injury. Furthermore, the treatment, as administered, was found to be detrimental to functional muscle recovery. Transcriptional profiling confirmed the pharmacological activity of pirfenidone, which attenuated fibrotic signalling by downregulating pro‐inflammatory cytokine and chemokine transcripts associated with excessive immune cell recruitment and exacerbated scar formation. While pirfenidone treatment modulated collagen architecture, as evidenced by altered fibre density, it failed to meaningfully decrease overall collagen content across histological and biochemical assessments. Moreover, the functional deficits observed here and in previous studies highlight a major knowledge gap in how systemic antifibrotics impact contractile recovery. Further exploration is warranted to ensure that treatments for fibrotic scarring do not inadvertently hinder the physiological recovery of VML‐injured muscle. In conclusion, given pirfenidone failed to provide a meaningful reduction in fibrosis and negatively affected functional outcomes, its potential as a clinical treatment for VML may be limited to specific adjunct applications aimed at immune modulation rather than primary scar reduction.

## AUTHOR CONTRIBUTIONS

Conceptualization: Jessica M. Motherwell, Christopher L. Dearth, and Stephen M. Goldman; Formal Analysis: Jessica M. Motherwell; Funding Acquisition: Christopher L. Dearth and Stephen M. Goldman; Investigation: Jessica M. Motherwell, Claudia E. Hernandez, Ondine Eken, and Stephen M. Goldman; Methodology: Jessica M. Motherwell, Christopher L. Dearth, and Stephen M. Goldman; Visualizations: Jessica M. Motherwell; Writing – Original Draft Preparation: Jessica M. Motherwell; Writing – Review and Editing: Jessica M. Motherwell, Claudia E. Hernandez, Ondine Eken, Christopher L. Dearth, Stephen M. Goldman. All authors have read and approved the final version of this manuscript and agree to be accountable for all aspects of the work in ensuring that questions related to the accuracy or integrity of any part of the work are appropriately investigated and resolved. All persons designated as authors qualify for authorship, and all those who qualify for authorship are listed.

## CONFLICT OF INTEREST

None declared.

## DISCLOSURES

The contents of this presentation are the sole responsibility of the author(s) and do not necessarily reflect the views, opinions or policies of Uniformed Services University of the Health Sciences (USUHS), The Henry M. Jackson Foundation for the Advancement of Military Medicine, Inc., the Department of Defense (DoD) or the Departments of the Army, Navy, or Air Force. Mention of trade names, commercial products, or organizations does not imply endorsement by the United States.

## GENERATIVE AI STATEMENT

Artificial intelligence (Gemini 3.5 Flash, 2026) was utilized solely as an editorial tool for language refinement during manuscript preparation, and all text was carefully reviewed to ensure the document accurately reflects the authors’ work and original voice.

## Data Availability

The datasets used and/or analysed during the current study are available from the corresponding authors upon a reasonable request.
